# Cervical Vertebral Body's Volume as a New Parameter for Predicting the Skeletal Maturation Stages

**DOI:** 10.1155/2016/8696735

**Published:** 2016-06-02

**Authors:** Youn-Kyung Choi, Jinmi Kim, Tetsutaro Yamaguchi, Koutaro Maki, Ching-Chang Ko, Yong-Il Kim

**Affiliations:** ^1^Department of Orthodontics, Biomedical Research Institute, Pusan National University Hospital, Gudeokro 179, Busan 602739, Republic of Korea; ^2^Department of Biostatistics, Clinical Trial Center, Pusan National University Hospital, Busan 602739, Republic of Korea; ^3^Department of Orthodontics, School of Dentistry, Showa University, Tokyo 1458515, Japan; ^4^Department of Orthodontics, School of Dentistry, University of North Carolina, Chapel Hill, NC 27599, USA; ^5^Dental Research Institute, Pusan National University Dental Hospital, Yangsan 626787, Republic of Korea; ^6^Institute of Translational Dental Sciences, Pusan National University, Busan 602739, Republic of Korea

## Abstract

This study aimed to determine the correlation between the volumetric parameters derived from the images of the second, third, and fourth cervical vertebrae by using cone beam computed tomography with skeletal maturation stages and to propose a new formula for predicting skeletal maturation by using regression analysis. We obtained the estimation of skeletal maturation levels from hand-wrist radiographs and volume parameters derived from the second, third, and fourth cervical vertebrae bodies from 102 Japanese patients (54 women and 48 men, 5–18 years of age). We performed Pearson's correlation coefficient analysis and simple regression analysis. All volume parameters derived from the second, third, and fourth cervical vertebrae exhibited statistically significant correlations (*P* < 0.05). The simple regression model with the greatest *R*-square indicated the fourth-cervical-vertebra volume as an independent variable with a variance inflation factor less than ten. The explanation power was 81.76%. Volumetric parameters of cervical vertebrae using cone beam computed tomography are useful in regression models. The derived regression model has the potential for clinical application as it enables a simple and quantitative analysis to evaluate skeletal maturation level.

## 1. Introduction

Among many skeletal maturity indicators, the hand-wrist radiographic analysis has been widely used [1–4]. However, this method of bone age determination requires additional exposure to radiation to obtain the data. Alternatively, skeletal age determination based on the cervical vertebrae from cephalometric films is used as this method does not require additional exposure to radiation [5, 6]. The cervical vertebral maturation (CVM) method classifies the stages of skeletal development upon the visual observation of the cervical vertebrae from the sagittal view. Baccetti et al. [6] modified and presented a simple method of determining the skeletal age from six developmental stages of the cervical vertebrae (C2–4); each developmental stage is a biological indicator indicating physical growth, especially the degree of mandibular growth. Unfortunately, the CVM method also presents several problems including the inconsistent changes in skeletal development during the growth period, a high level of intra- and interobservation error during the tracing activities of lateral cephalometric radiography, and inaccurate measurements of bone mass [7].

To improve the limitations of the methods mentioned above, Chen et al. [8] developed a quantitative analytical method from the sagittal view and presented an objective indicator replacing the lateral cephalometric radiography. Yang et al. [9] also performed quantitative shape analysis from the axial view of the cervical vertebrae. The study concluded that the shape analysis enhances the ability to explain bone maturation as opposed to relying only on chronological age. The axial shape of the cervical vertebrae can serve as a biological indicator of ossification. These studies reported the regression models for skeletal age estimation from the lateral and axial images of the cervical vertebral shape, respectively.

However, the growth of the human body is not two-dimensional (2D); there is a limit to which 2-dimensional approach cannot fully describe elements and factors involved in the growth of human body. The growth of the human body occurs in three dimensions and sequence. Growth in width occurs first, followed by growth in the anteroposterior dimension and, lastly, growth in height [10, 11]. The growth of the maxillary skeletal width concludes before the peak pubertal growth; the growth in anteroposterior dimension and height continues throughout puberty [12]. Therefore, a three-dimensional (3D) approach is required to evaluate cervical vertebrae maturation. Previous studies used cone beam computed tomography (CBCT) in limited 2D applications, even though CBCT enables the observation of 3D multiplanar images from the head and neck area including the vertebrae. Thus, there is still a need to study 3D CVM assessment.

Therefore, the objective of this study is to determine the correlation of the volume parameter derived from 3D images of the second, third, and fourth cervical vertebrae using CBCT with skeletal maturation stage and to propose a new formula for predicting skeletal maturation using multiple regression analysis.

## 2. Material and Methods

### 2.1. Subjects

This study involved a retrospective review of available data. The sample population consisted of 54 women and 48 men (total: 102, range: 5–18 years, and mean age: 9.9 years) as determined by the inclusion criteria established by the Department of Orthodontics, Pusan National University Hospital, and based on the available hand-wrist radiographs and CBCT images. Data was obtained for orthodontic treatment (impacted teeth and craniofacial and dental trauma cases) and skeletal maturation evaluation. Investigators excluded patients diagnosed with a congenital or postnatal malformation or syndrome, growth impairment mental retardation, or preexisting conditions with potential impact on the vertebrae or hand-wrist development from the sample population (Table 1). This study was reviewed and approved by the Institutional Review Board of Pusan National University Dental Hospital (PNUDH-2014-019).

### 2.2. Skeletal Maturation Level Assessments

To assess skeletal maturational stages from hand-wrist radiographic films, investigators used the Sempé maturation level (SML, 0–999) method [13]. Investigators obtained the hand-wrist radiographic films from a Rotanode DRX-3724HD (Toshiba Medical Systems, Tokyo, Japan) according to the exposure parameters of 42 kVp, 100 mÅ, and 0.032 seconds. The SML method quantifies the skeletal maturation level into an index ranging from 0 at birth to 999 at the stage when growth is complete, on a continuous scale, and provides a more refined ossification level. One investigator (CYK) assessed SML for all patients. For validating the intrainvestigator's error, all samples were reassessed twice at two-week intervals.

### 2.3. Volume Measurements

For the volume measurements, investigators used the CBCT dataset. Investigators recorded CBCT scans (CB MercuRay (Hitachi Medical, Tokyo, Japan)), with the subject in an upright position for maximum intercuspation. The Frankfurt horizontal (FH) plane was parallel to the floor. Investigators used the CBCT settings of 100 kVp tube voltage, 10 mÅ tube current, 9.6 seconds' scan time, 192.5 mm diameter spherical field of view (FOV), and 0.376 mm voxel size. Investigators reconstructed the scans with the 3D image software OnDemand3D (Cybermed Co., Seoul, Korea) by the same gray-scale condition (window width 4000, window level 1000). Vertebral bodies of the second, third, and fourth cervical vertebrae were segmented and built in a separate 3D image.

**Figure 1:**
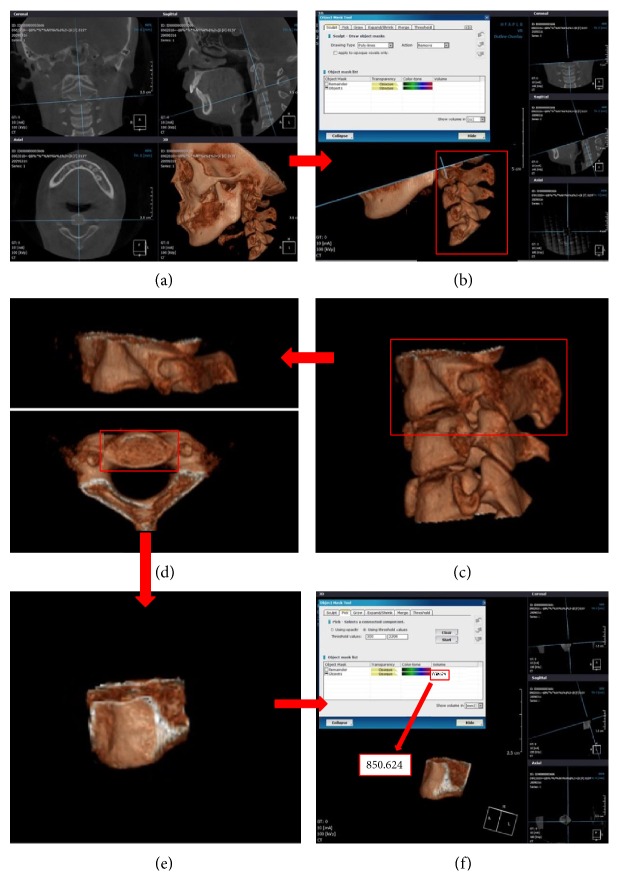
Volume measurement process. CBCT image isolated from each cervical vertebra (C2–C4). (a) The initial full field image. (b)–(e) “Remove” function on manual segment tool was in progress. (f) Finally, the volume of each cervical vertebra was obtained.

Investigators followed these segmentation procedures. First, the investigators obtained the bone image with presettings to achieve the window width level (1180) and uniform opacity threshold (opacity threshold: 329~2124). Second, the investigators used the segmentation tool to delete the opacity area corresponding to skin and airway and left the second, third, and fourth cervical vertebrae bones from the vertical view from the sagittal CVM image. Finally, the investigators built 3D images only with the vertebral body with the transverse arch removed from each cervical vertebral image. For the second vertebra, the dentocentral synchondrosis was utilized as the basis to segregate the odontoid process from the body. After that, each vertebral body area was designated from the 3D image using the “pick” function, with volume measured in cubic millimeters (mm^3^) (Figure 1).

### 2.4. Statistical Analysis

The same investigator (CYK) repeated each volumetric parameter after two weeks. Investigators used Pearson's correlation coefficient analysis to demonstrate the association between the skeletal maturation index (SMI) and the SML. To use the SMI, the investigators replaced the SML as an indicator representing skeletal maturity. Each volumetric parameter was subjected to Pearson's correlation coefficient analysis to understand the correlation between each parameter and SML. In addition, the investigators conducted a simple regression analysis to understand the explanation power of the SML through the volumetric parameters at each of the cervical vertebrae and the sex-related interaction. We used SPSS 21.0 (SPSS, Chicago, Il, USA) to analyze the data with a *p* value less than 0.05. We evaluated the intrainvestigator's reliability and reproducibility for 20 randomly selected subjects after two weeks.

## 3. Results

### 3.1. Skeletal Maturation Assessment

The sample population of 54 women and 48 men (total: 102, range: 5–18 years, and mean age: 9.9 years) exhibited minimum skeletal maturation (Sempé maturation level) with a range of 14.5% to 98.5% (Table 1). Pearson's correlation coefficient analysis demonstrated an association between the SMI and the SML. To use the SMI, investigators replaced the SML, as the indicator representing skeletal maturity. The association between the two was 0.950, which is considered a very high correlation, and as a result, the SML was used as the skeletal maturity indicator.

### 3.2. Correlation between Cervical Vertebral Body Volumes and Skeletal Maturation Level

The regression equation for the individual vertebral body volume for the SML was built using simple regression analysis. The fourth cervical vertebra had the highest and the second cervical vertebra had the lowest correlation between each vertebral body and the SML, respectively. The second cervical vertebra did not represent a proportional increase in the SML in men and women. As the volume level increased, it demonstrated a rapid increase in the SML in volumes in the range of 2000~3000 mm^3^ in men (Table 2). Consequently, there was a significant difference in the level of volume change between men and women; the regression curve for men was not a linear line but rather a secondary curve. The explanation power of 60.47% is considered relatively low compared to the regression equations for the other parameters.

The third cervical vertebra demonstrated a linear regression curve with a proportional increase in the SML as each volume level increased in both men and women. However, the increase was greater in men, presenting a steeper slope. The explanation power of the third cervical vertebral body volume was 81.29%.

**Figure 2:**
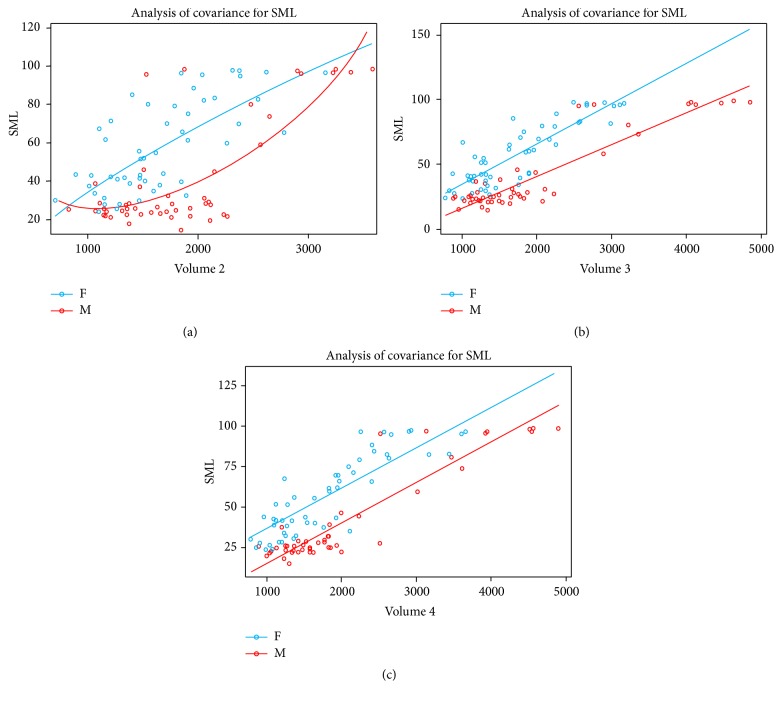
Estimated skeletal maturation level within each volume level of the second, third, and fourth cervical vertebrae. (a) A quadratic regression model of the second cervical vertebra. (b) Linear regression model including sex-related interaction of the third cervical vertebra. The degree of increase differs between women and men. (c) Linear regression model of the fourth cervical vertebra. The degree of increase is the same for women and men.

The fourth cervical vertebra demonstrated a proportional increase in the SML as the volume increased in both men and women, with a somewhat similar increase. Consequently, a linear regression curve with a similar slope was present in both men and women. The regression equation was (SML) = −11.779 + 0.026 × C4_volume + 22.010 × sex_F, with an explanation power of 81.76% (Table 3). The estimated SML within each volume level of the second, third, and fourth cervical vertebrae are in Table 2 and Figure 2.

## 4. Discussion

Several studies have utilized regression formulas using the lateral aspect of the cervical vertebral bodies to estimate the status of cervical vertebrae growth [8, 14–16]. However, there are limitations to these previous studies as most determined human growth using 2D data, whereas human growth changes in three dimensions. The cervical vertebrae also change three-dimensionally at the growth phase through a modeling process in a particular area. Therefore, the 3D approach to evaluating skeletal maturation is needed. In this study, to attempt the volumetric evaluation of skeletal age, we used CBCT. It is possible to execute volumetric analysis, much more than the measurement of the length and angle of the boundary of 2D analysis for the craniofacial region [17, 18]. However, concerns remain about whether CBCT imaging on top of routine 2D radiography is necessary for every patient or if the additional imaging is only necessary for special circumstances (e.g., craniofacial syndrome, impacted teeth) with the use of a suggested analytical method of CBCT. We obtained the CBCT data in this study following the “as low as diagnostically acceptable (ALADA)” principle. And we believe that as CBCT develops, CBCT can replace conventional panorama and cephalometric radiograph. Furthermore we expect CBCT with lower radiation. We would like to emphasize that the intent of the study was to use the acquired data more valuably.

From the results, each cervical vertebra was examined for an increase in SML as volume increased. When examining the correlation between each cervical vertebra's volume and the SML, the fourth cervical vertebra showed the highest correlation while the second cervical vertebra showed the lowest correlation. In the second cervical vertebra, investigators demonstrated that the increase in SML, as a result of an increase in volume, was not consistent. In most cases, women have higher values in their volume level. However, the increase in SML in accordance with the increase in volume shows a difference between men and women. In women, the change in the SML in accordance with the volume level was relatively consistent throughout the process; a rapid increase in the SML increased the volume level. This tells us that the volume of the second cervical vertebra in men changes drastically during the pubertal growth peak. There is no significant difference between men and women before the pubertal growth peak when the volume is 1000 mm^3^ and near the completion of pubertal growth peak with a volume of 3000 mm^3^. There is a significant difference between men and women when the volume is in the range of 1500–3000 mm^3^. However, the explanation power of the regression model using the second cervical vertebra is 60.5%, which is lower than that reported in previous studies [15, 16].

The third cervical vertebra exhibited a different result; there was a significant difference between men and women in all areas, resulting in a higher SML in women at all volume levels. Furthermore, there was a difference in the slope of the regression coefficient for men and women, in that the SML increased more rapidly in women as the volume of the third cervical vertebra increased. However, it is well known that increases in the SML are relatively consistent as the volume increased in both men and women. The explanation power of 81.3% for the SML from the third cervical vertebra is similar to previously published studies.

Lastly, we observed that the change in the SML as the volume increased in the fourth cervical vertebra was relatively consistent in all areas/regions, like the third cervical vertebra. There was a significant difference between men and women at each volume level, and the SML was higher in women. However, unlike the third cervical vertebra, there was no difference in the regression coefficient in men and women, which in turn indicates that the SML increased consistently as the cervical vertebral volume increased. Furthermore, it also demonstrated an explanation power of 81.8% for the SML, representing the highest of the three cervical vertebrae.

The changes in the volume of the cervical vertebrae observed in our study are similar to that observed by Crawford et al. [19]. Crawford et al. stated that changes in volume are due to active bone remodeling in C2 that occurs more than it does in C3 [19]. In contrast, there is less resorption of preformed bone in C3, resulting in more consistent growth in volume. Altan et al. [20] conducted a study in girls and reported a similar outcome in that the growth in C2 exhibited twice the amount of growth in C3 in the SML of a 14.5-year-old. After this age, growth starts to diminish, a finding confirmed when comparing the identical growth of the SML to a 16.5-year-old. This, in turn, demonstrates that C3 exhibits a more consistent change in volume during the growth period.

Furthermore, the explanation power for the SML, based on the change in volume, was relatively high. This finding is similar to or higher than the estimated outcome by using the 2D or 3D length parameters [8, 9, 14–16]. The cervical vertebral maturation index, a tool first suggested by Lamparski to replace the hand-wrist radiograph, segregates the growth stage in six steps using the second, third, and fourth cervical vertebrae [16]. O'Reilly and Yanniello [21] determined that there is a direct correlation between the CVM stage and mandibular growth using this method; this is useful when evaluating growth in orthopedic treatment. In addition to this, this technique does not require additional irradiation for a hand-wrist radiograph. In 2002, Baccetti et al. [6] were able to reduce Lamparski's process to four steps with a modified CVM. However, the second, third, and fourth cervical vertebrae had to be observed. This is a problem as there is significant error among the test subjects, which is hard to quantify [6]. To complement these drawbacks, a study was conducted to estimate the CVM in a quantitative way [8, 9, 14–16], there were a number of difficulties in the clinical application as the study had to include most of the C2–C5 vertebrae, and/or it was deemed onerous to measure many parameters in each cervical vertebra. However, the present study enabled the estimation of the SML by measuring the volume of C4 alone in a similar way. This allows the use of a skeletal maturation estimation method that is much more simple and accurate for use in clinical application.

The purpose of this study was to suggest an objective and quantitative method to estimate growth using the 3D volumetric analysis of cervical vertebrae by 3D CBCT images instead of the existing 2D CVM images. It is important to establish a method to estimate the SML by measuring 3D volume from CBCT imaging in patients at their growth phase, before orthopedic treatment, and to establish multiple regression models. This method measures fewer cervical vertebrae and associated parameters compared to the current CVM method. Using the 3D volumetric analysis of cervical vertebrae enables convenient clinical application, as well as a quantitative and accurate evaluation of the results

## 5. Conclusions

CBCT enables the measurement of the volume of cervical vertebrae, which is difficult, in addition to the sagittal image observed through the existing 2D radiography. In this study, a multiple regression model for the SML was established based on the volumes of the second, third, and fourth cervical vertebral bodies measured using CBCT. In particular, a higher explanation power for the SML was achieved through the linear regression model using the volume in the fourth cervical vertebral body, compared to that from the models in previously published studies [15, 16]. Therefore, the derived multiple regression model has potential clinical application as it enables a simple and quantitative analysis to evaluate the SML in comparison to the existing CVM evaluation methods.

## Figures and Tables

**Table 1:** Descriptive characteristics of the subjects.

Sample size (*n* = 102)	Boys (*n* = 48)		Girls (*n* = 54)	
	Chronologic age	Sempé maturation level (SML, %)	Chronologic age	Sempé maturation level (SML, %)
Mean	9.88	40.86	9.96	56.45
Max	18.00	98.50	18.00	97.60
75% quartile	11.50	44.80	11.00	79.50
Median	8.00	26.15	9.00	51.70
25% quartile	8.00	23.00	8.00	37.30
Min	5.00	14.50	6.00	23.80

**Table 2:** Estimated skeletal maturation level within each volume level of the second, third, and fourth cervical vertebrae.

Volumes (mm^3^)		1000	1500	2000	2500	3000	3500	*R*-square
2nd vertebra (C2) (quadratic regression model)	Female	33.994	52.632	69.358	84.170	97.070	100>	0.6047
	Male	26.803	28.076	37.237	54.285	79.220	100>	
	Diff.	7.191	24.556	32.121	29.886	17.850	—	
	*P* value	0.349	<0.0001	<0.0001	<0.0001	0.121	—	

3rd vertebra (C3) (linear regression model including sex-related interaction)	Female	34.387	50.041	65.695	81.349	97.004	100>	0.8129
	Male	16.485	28.715	40.945	53.176	65.406	77.636	
	Diff.	17.903	21.327	24.750	28.174	31.598	35.021	
	*P* value	<0.0001	<0.0001	<0.0001	<0.0001	<0.0001	<0.0001	

4th vertebra (C4) (linear regression model)	Female	35.761	48.527	61.292	74.057	86.823	99.588	0.8176
	Male	13.751	26.517	39.282	52.047	64.812	77.578	
	Diff.	22.010	22.010	22.010	22.010	22.010	22.010	
	*P* value	<0.0001	<0.0001	<0.0001	<0.0001	<0.0001	<0.0001	

**Table 3:** The results of multiple regression analysis using the *R*-square selection method.

Multiple regression models	Independent variables	Parameter estimate	Standard error of estimate	*t* value	*R*-square
4th-vertebra volume	Intercept	−11.779	3.155	−3.73	0.818
	C4_volume	0.026	0.001	19.80	
	Sex_F	22.010	2.366	9.30	
